# Application of Ureteroscope in Emergency Treatment with Persistent Renal Colic Patients during Pregnancy

**DOI:** 10.1371/journal.pone.0146597

**Published:** 2016-01-11

**Authors:** Shilin Zhang, Guoqing Liu, Yongfu Duo, Jianfeng Wang, Jierong Li, Chunjing Li

**Affiliations:** Department of Urology, Foshan Maternal and Child Health Hospital, Southern Medical University, People's Road No. 11, Foshan City, Guangdong Province, 528000, China; University of Malaya, MALAYSIA

## Abstract

**Background:**

Although the application of ureteroscopy in the treatment of ureteral calculi during pregnancy has been on the rise, for persistent renal colic patients without ultrasound-detected ureteral calculi, it may represent a clinical dilemma due to the potential risks for both mother and fetus.

**Objective:**

The aim of the present study is to present our experience with the application of the ureteroscope in the emergency treatment of persistent renal colic patients during pregnancy.

**Methods:**

From March 2009 to September 2014, a total of 117 pregnant women who received ureteroscopy for persistent renal colic were retrospectively analyzed. Patients were divided into three groups according to duration of the persistent renal colic: Group A (within 12 hours; 24 cases); Group B (12 to 24 hours; 76 cases); and Group C (more than 24 hours; 17 cases). The stone-free rate, complications, and other qualitative data were analyzed.

**Results:**

Of the 117 patients, 31 patients who were found not to have renal or ureteral calculi received ureteroscopic double-J (DJ) stent insertion, whereas 86 patients who were found with ureteral calculi received ureteroscopic lithotripsy (URSL) and DJ stent insertion. Among them, 24 patients (27.9%) were found with ureteral calculi by ureteroscopy rather than ultrasound. In addition, 73 patients (84.9%) had complete fragmentation of calculi; 12 patients (10.3%) had a threatened abortion (the rates of threatened abortion in Groups A, B and C were 8.3% vs. 6.5% vs. 29.4%; Group C compared with Groups A and B, *p*<0.05), and one patient (1.2%) had urosepsis (in Group C). However, these complications were cured with conservative treatment, without postpartum infant and maternal complications.

**Conclusion:**

For pregnant patients with persistent renal colic/ureteral calculi and hydronephrosis, ureteroscopic DJ stent insertion and URSL are effective and safe options when conservative treatment fails, even if no urinary calculi were found by ultrasound. At the same time, for patients with persistent renal colic during pregnancy, early application of ureteroscopy may reduce the risk of preterm birth.

## Background

With the high frequency of urolithiasis and a higher likelihood of women developing kidney stones in pregnancy, the diagnosis and treatment of urolithiasis during pregnancy are very frequent [1]. The incidence of urinary calculi during pregnancy is approximately 0.026–0.531% and may be associated with 40% of preterm births [2, 3, 4]. Ureteroscopy is often considered the first definitive treatment of obstructive ureteral calculi during all trimesters of pregnancy [3], but for persistent renal colic patients without ultrasound-detected ureteral calculi, it still remains a difficult condition concerning both diagnosis and treatment. Using pain symptoms such as back pain radiating to the side, lower abdomen or genital area, we can make a preliminary diagnosis of renal colic. However, the most important pain symptom is a knocking pain in the kidney area. Although KUB, IVU, and CT scan are considered useful in evaluating an individual with renal colic [5, 6, 7, 8], these image scans should be avoided during pregnancy due to the teratogenesis risks associated with radiation exposure [1]. Therefore, abdominal ultrasound examination is the preferred and decisive method of evaluation for a pregnant woman with abdominal pain and/or suspected renal colic [9]. However, although it has a high specificity for the diagnosis of ureteral lithiasis, the sensitivity of this method is very low [10]. For persistent renal colic patients without ultrasound-detected ureteral calculi, this low sensitivity may present a clinical dilemma for both diagnosis and treatment. The aim of the present study was to assess the safety and effectiveness of ureteroscopy as a useful diagnosis and treatment option in women presenting persistent renal colic/ureteral calculi during pregnancy.

## Patients and Method

Between March 2009 and September 2014, 128 pregnant women with persistent renal colic/ureteral calculi requiring active therapy were referred to our institution. Written informed consent was obtained from all individuals before ureteroscopy. The protocol was approved by the Ethics Committee of Foshan Maternal and Child Health Hospital of Southern Medical University. The main inclusion criterion for participating patients was presenting persistent renal colic/ureteral calculi and hydronephrosis. Of them, cystoscopic double-J (DJ) stent insertion was performed in 5 patients, and percutaneous nephrostomy was applied in 6 patients. Severe patients presented with definite urinary tract infection associated with fever, and 4 were suspected to be at risk of preterm birth. The remainder of the 117 pregnant women who were included in our retrospective study received invasive ureteroscopy. Patients were divided into three groups according to the duration of intermittent or continuous intractable/recurrent renal colic: Group A consisted of patients who experienced persistent renal colic for 12 or fewer hours (n = 24); Group B consisted of patients who experienced persistent renal colic for a duration 12 to 24 hours (n = 76); and Group C consisted of patients who experienced persistent renal colic for more than 24 hours (n = 17). Invasive ureteroscopy included ureteroscopy (URS) and/or URSL, and DJ stent insertion. URS was performed under general (n = 72) or spinal (n = 45) anesthesia via a 9.5F semirigid ureteroscope (Karl Storz, Tuttlingen, Germany) with a sliding guide wire (0.035 mm) or with guidance of a ureteral catheter (4-6F) during the first 2 trimesters and last trimester of pregnancy (48 and 11 patients, respectively), and the flexible uteroscope (Olympus URF-V) in the last trimester of pregnancy (16 patients). The average patient age was 25.5±4.6 years (range 16–41 years), and the gestation period varied from 9 to 36 weeks. The assessment included the general situation, blood tests, urinalysis, urine culture, renal function, blood culture if necessary, ultrasound, duration of pain from onset to surgery, duration of the operation and consultation of the Department of Gynecology. No KUB, IVU, or CT scan was performed for any patient to avoid damage to the fetus. All patients had received preoperative conservative treatment. No patients underwent ureteral dilation. The stones were fragmented with pneumatic ballistic lithotripsy or Ho:YAG laser. DJ stents were routinely placed in all patients. The postoperative follow-up included obstetric care aiming to ensure maternal and fetal health as well as outpatient urological follow-up represented by clinical assessment, ultrasound examination, urinalysis, and urine culture. DJ stents was removed 4 weeks or so after the USL, but patients who did not undergo removal of calculi were retained for longer periods of time, until the postpartum period. A summary of clinical characteristics and surgical information about the patients is listed in Table 1(Details see S1 Dataset). The chi-square test was used to evaluate and compare stone-free status, assessment of clinical indicators, follow-up assessment, time of renal colic duration and operation, postoperative follow-up procedures, and complications. Fisher’s exact test was used to analyze qualitative data. A p-value of less than 0.05 was considered to be statistically significant.

**Table 1:** Summary of main clinical characteristics and surgical information about persistent renal colic patients during pregnancy. **Group A:** patients who experienced refractory renal colic for 12 or fewer hours. **Group B:** patients who experienced refractory renal colic duration within 12 to 24 hours. **Group C:** patients who experienced refractory renal colic for more than 24 hours. ※: Compared with Groups A and B, the difference is statistically significant (*p*<0.05)

Items	Group A	Group B	Group C
**【The number of cases】**	24	76	17
**【Cases with ureteral calculi】**	16	57	13
**【Cases of no found ureteral calculi】**	8	19	4
**【Positive urine culture】**	3	7	3
**【Microscopic hematuria】**	24	76	16
**【Leukocyturia】**	8	21	8
**【Irritative voiding symptoms】**	5	11	6
**【The duration of the operation (minutes)】**	27±4.8	31±4.5	22±6.8
**【Fever】**	0	4	1
**【Stone-free】**	14	49	10
**【Stone fragments migrated retrograde to the renal pelvis】**	2	8	3
**【Urosepsis】**	0	0	1
**【Uterine contractions】**	2	5	5 ※
**【Ureteral perforation】**	Null	Null	Null
**【Serious obstetric, fetal or urologic complications】**	Null	Null	Null

## Results

A total of 117 patients (100%) had hydronephrosis (1st degree hydronephrosis in 66, 2nd degree in 39, and 3rd degree in 12). Among 86 patients found with ureteral calculi, an ultrasound image of obstructive ureteral calculi was obtained in 62 patients (72.1%) with a mean stone diameter of 8.2±0.6 mm. Meanwhile, another 24 patients (27.9%) were found with ureteral calculi just by ureteroscopy. Thirty-one patients received ureteroscopic DJ stent insertion when no ureteral calculi were found, whereas 86 patients received URSL with holmium laser or pneumatic ballistic; all patients received postoperative DJ stent insertion. Of the 86 patients with ureteral calculi, 73 patients (84.9%) had complete fragmentation of calculi. The stone-free rates were 87.5%, 86.0% and 77.0% in Groups A, B and C, respectively (*p*>0.05). The stone fragments retrograde migrated to the renal pelvis in 13 patients (15.1%, 13 of 86). Twelve patients (10.3%, 12 of 117) had a threatened abortion or suspicion of threatened abortion (the rates of threatened abortion in Groups A, B and C were 8.3% vs. 6.5% vs. 29.4%; when Group C was compared with Groups A and B, the difference was statistically significant, *p*<0.05). One patient (1.2%) had urosepsis (in Group C), but these problems were resolved immediately using conservative treatment. All of patients completed pregnancy to full term, and no other serious obstetric, fetal or urologic complications were observed. The remaining 31 patients (29.9%) with renal colic, although presenting with hydronephrosis, were not found to have any calculi by ureteroscopy or ultrasound examination. Twenty-two women had irritative voiding symptoms; five had fever. Different levels of microscopic hematuria were observed in 116 patients, leukocyturia in 37 patients, and positive urine culture in 13 patients.

## Discussion

Persistent renal colic during pregnancy is a true urological emergency that can become life-threatening sepsis and have associated complications for both the mother and fetus [4]. Thorough urological, gynecological and general surgical evaluations are needed to exclude other surgical emergencies such as acute appendicitis and a twisted ovarian cyst [11]. Existing research shows that maternal kidney stones are significantly associated with several pregnancy complications, including recurrent abortions, hypertensive disorders, gestational diabetes, and cesarean deliveries [12]. Although ordinary X-ray, IVU and CTU are convenient and conclusive methods of diagnosis in non-pregnant urologic patients, they are not used routinely in symptomatic pregnant women because ionizing radiation is potentially teratogenic and its effects have not been well defined [4, 13]. Abdominal ultrasonography was the preferred and most decisive evaluation method for renal colic during pregnancy in many reports and in our series. However, although it has a high specificity of 90% for the diagnosis of ureteral lithiasis, the sensitivity of this method is quite low (11%-24%) [10]. Atar M, et al., using the renal resistive index for managing symptomatic persistent hydronephrosis during pregnancy, found that there was a significantly higher mean resistive index in the kidneys with ureteral obstruction than in the contralateral normal kidneys [14]. In our series of 86 patients who were found to have ureteral calculi, an ultrasound image of ureteral calculi was obtained in 62 patients (72.1%), and 24 patients (27.9%) were found to have ureteral calculi by ureteroscopy rather than ultrasound. This finding shows that ultrasonography can be used as a decisive confirmation method for renal colic patients during pregnancy and that ureteroscopy can be used as an auxiliary measure. MRI is a valuable and well tolerated diagnostic for evaluating painful hydronephrosis in pregnancy. It has a distinguished ability to differentiate between pathological and physical obstructions. However, small calculi were only identified using high resolution T2-weighted magnetic resonance imaging [15].

For patients with renal colic and ureteral calculi during pregnancy, 20% to 30% will ultimately need some form of active treatment [16]. Indications for intervention are largely based on individual patient factors and generally include uncontrolled pain, sepsis, progressive hydronephrosis, obstructed solitary kidney, bilateral obstruction, obstetric complications such as premature labor onset and pre-eclampsia and poor access to urological care/equipment [17]. A number of techniques are used for stone extraction, including USL, basket extraction and the lithoclast [18]. Inserting a ureteral catheter, percutaneous nephrostomy (PCN) or ureteroscopy currently represent the main treatment options for ureteral calculi during pregnancy. However, for persistent renal colic patients without ultrasound-detected ureteral calculi, the colic may represent a clinical problem due to the potential risks for both the mother and fetus. The management of ureteroscopy for the treatment of ureteral calculi during pregnancy has been increasing. Technological advancements such as the development of the semi-rigid or flexible ureteroscope, improvements in the design of baskets used for retrieval, and the availability of lasers have enabled the atraumatic fragmentation of stones [19]. The physiologic dilatation of the ureter that stems from hormonal and mechanical factors facilitates the use of the ureteroscope in pregnant women [20]. In our series, no patients were treated with ureteral expansion. Furthermore, the recent development of mini–ureteroscopes, stone fragmentation tools and safe anesthesia methods during pregnancy has made it possible to successfully access and treat any stone within the upper tract in a relatively atraumatic manner [21]. Some authors think ureteroscopy may be considered a safe and effective first-line definitive therapeutic option in pregnant patients requiring intervention for stone disease [22, 23]. In our series, 24 patients (27.9%) were found with ureteral calculi just by ureteroscopy. Thus, first of all, ureteroscopy can be an effective supplement to diagnose ureteral calculi during pregnancy. As renal colic and ascending urinary tract infections can become life-threatening sepsis and have associated complications for both the mother and fetus, early interventions are more likely to achieve better outcomes. In our cohort, 31 patients with renal colic and hydronephrosis were not found to have renal and ureteral calculi by ultrasound and ureteroscopy. They also underwent DJ tube indwelling through ureteroscopy and quickly experienced pain relief and subsequent hydronephrosis without the occurrence of urinary and obstetric complications. Renal colic during pregnancy is not necessarily caused by kidney or ureteral calculi. Some hydronephrosis cases may be caused by enlarged uterus oppression. Furthermore, for renal colic patients without ureteral calculi, a ureteral microscopy check and ureteral DJ stents can help relieve obstruction. These methods are also effective in reducing pain and promoting drainage in hydronephrosis.

In these cases, DJ stents were routinely inserted in all patients after ureteroscopy. Finney et al. first described the DJ stent in 1978 [24]. However, for non-pregnant women with ureteral calculi, the use of a DJ stent during URSL is controversial [25, 26]. As the most common complication of stent insertion is represented by encrustation, some researchers suggest that stents placed during the first trimester should be changed on a monthly basis [16]. Meanwhile, urinary tract infections, stent migration and other complications of internal stent placement have been described [16, 25, 27]. As a consequence of these difficulties, Denstedt and Razvi suggested that a ureteral stent should be reserved for the later stages of pregnancy [27]. Complications associated with the use of DJ stents are basically related to the stent material. With the improvement of tube materials, there are some DJ tubes that can indwell for 6–12 months or longer. In our series, although individual patients presented with urinary tract symptoms and microscopic hematuria, the symptoms were tolerated, and there were no obvious effects on the fetus and mother.

Contraindications of the use of ureteroscopy during pregnancy include a lack of ureteroscopy experience, inadequate equipment, large or multiple stones, graft or solitary kidney, kidney damage, and sepsis. Notably, using low-pressure perfusion and short operation times are recommended for the best outcome. In contrast to non-pregnant urologic patients, the primary purpose of treatment is not to completely remove urinary stones but to achieve unobstructed drainage. Although there is an incidence of obstetric complications such as uterine contraction, the principle of prudence in pregnant patients undergoing ureteroscopy is underlined; at the same time, because persistent renal colic itself may also induce contractions, we need to perform a comprehensive evaluation of all options. In our series, the patient with severe postoperative complications (urosepsis) is the one who encountered intermittent pain for more than 24 hours. The pain was resolved immediately using conservative treatment, and she completed the pregnancy to full term. No serious obstetric or fetal complications were observed in our cohort. We speculate that early ureteroscopy and DJ tube insertion, resulting in drainage and pain relief, may be conducive to the recovery of patients and may reduce complications. Of course, there are many possible causes for serious postoperative complications, including operation duration, perfusion pressure, and proficiency of the surgeon. In the treatment of threatened abortion, the patient needs to be given strong support and reassurance from the obstetrician. Despite some authors suggesting a consistent awareness of the potential risk of ureteral perforation, there were no occurrences of perforation in our series.

This study is limited by the nature of retrospective analyses and a relatively small cohort. However, the results of this study suggest that for pregnant patients with persistent renal colic/ureteral calculi and hydronephrosis, ureteroscopy, DJ stent insertion and URSL are effective and safe options when conservative treatment fails, even in the absence of ultrasound-detected urinary calculi. These methods may also reduce the risk of premature births. Nevertheless, the necessity of stone clearance during pregnancy cannot be overemphasized, with an emphasis on ensuring that the urinary tract becomes unobstructed quickly to reduce the occurrence of complications such as abnormal uterine contractions or premature birth and abortion.

## Supporting Information

S1 Dataset Raw data of the 117 pregnant women who received invasive. (XLS)
